# Experimental Pandemic (H1N1) 2009 Virus Infection of Cats

**DOI:** 10.3201/eid1611.100845

**Published:** 2010-11

**Authors:** Judith M.A. van den Brand, Koert J. Stittelaar, Geert van Amerongen, Marco W.G. van de Bildt, Lonneke M.E. Leijten, Thijs Kuiken, Albert D.M.E. Osterhaus

**Affiliations:** Author affiliations: Erasmus Medical Centre, Rotterdam, the Netherlands (J.M.A. van den Brand, K.J. Stittelaaar, G. van Amerongen, M.W.G. van de Bildt, L.M.E. Leijten, T. Kuiken, A.D.M.E. Osterhaus);; ViroClinics Biosciences BV, Rotterdam (K.J. Stittelaar, G. van Amerongen, A.D.M.E. Osterhaus)

**Keywords:** Pandemic (H1N1) 2009, influenza, viruses, cats, pathogenesis, transmission, pathology, dispatch

## Abstract

To demonstrate that pandemic (H1N1) 2009 virus may cause respiratory disease in cats, we intratracheally infected cats. Diffuse alveolar damage developed. Seroconversion of sentinel cats indicated cat-to-cat virus transmission. Unlike in cats infected with highly pathogenic avian influenza virus (H5N1), extrarespiratory lesions did not develop in cats infected with pandemic (H1N1) 2009 virus.

Soon after pandemic (H1N1) 2009 virus emerged in North America, infections in domestic cats were reported (*1**,**2*). Infection with highly pathogenic avian influenza (HPAI) virus (H5N1) leads to severe and often fatal diffuse alveolar damage and systemic virus spread in cats (*3**–**5*). In contrast, seasonal human influenza viruses do not cause disease in cats (*6*). To elucidate the pathogenesis of pandemic (H1N1) 2009 virus infection in cats, we studied 8 laboratory cats intratracheally infected with this virus.

## The Study

Pandemic (H1N1) 2009 virus (A/Netherlands/602/2009) was isolated from a 3-year-old girl from the Netherlands who had mild influenza after she visited Mexico in early 2009. Virus was cultured in embryonated chicken eggs and passaged once in MDCK cells (*7*).

We used 2 groups (4 cats/group) of 16-week-old, purpose-bred, specific pathogen–free, European shorthair cats that were seronegative for hemagglutination-inhibition (HI) antibodies against pandemic (H1N1) 2009 virus and circulating seasonal influenza A viruses. These cats were intratracheally infected with a 10^6.0^ tissue culture infectious dose (TCID_50_) of pandemic (H1N1) 2009 virus. A third group of 3 sentinel cats were housed with these 2 infected groups (1 with group 1 and 2 with group 2) from 2 days postinfection (dpi) onward. Serum samples were obtained on 0, 4, 7, and 21 dpi and stored at –20°C until tested for HI antibodies against pandemic (H1N1) 2009 virus (*8*).

All 11 cats were monitored daily for clinical signs, and body temperature was measured at 15-min intervals. Nasal, pharyngeal, and rectal swab specimens were obtained daily from all cats. After being anesthetized with ketamine, all cats were killed by exsanguination. Cats in groups 1 and 2 were killed at 4 dpi and 7 dpi, respectively. Sentinel cats were killed at 21 dpi. Experiments were performed under BioSafety Level 3 by using protocols approved by our Institutional Animal Welfare Committee.

Necropsies were performed according to a standard protocol. Lung, nasal turbinate, nasal septum, larynx, trachea, bronchus, tracheobronchial lymph node, nictitating membrane, tonsil, heart, liver, spleen, kidney, pancreas, duodenum, jejunum, colon, adrenal gland, brain, and olfactory bulb samples were obtained, were fixed in formalin, and processed to obtain sections for staining with hematoxylin and eosin.

For detection of viral antigen, tissue sections were stained with viral nucleoprotein–specific antibody (*6*). Alveolar epithelial cells were phenotyped by using a destaining–restaining technique (*9*). After organ samples were weighed and stored at –80°C, infectious pandemic (H1N1) 2009 virus was quantified by limiting dilution virus isolation in MDCK cells (*10*).

Cats in groups 1 and 2 infected with pandemic (H1N1) 2009 virus showed mild-to-moderate clinical signs (lethargy, appetite loss, rapid and labored breathing, and protruding nictitating membrane) after 1 dpi or 2 dpi onwards. Average body temperatures increased after 1 dpi, showed a maximum increase of ≈1.5°C by 2 dpi, and returned to baseline values within 4–5 dpi (Figure). Sentinel cats showed no clinical signs. Two cats in group 1 (2 pharyngeal samples) and 2 cats in group 1 and 1 cat in group 2 (1 pharyngeal sample) had low virus titers during 1–4 dpi (<10^1.8^ TCID_50_/g). Nasal swab specimens from all sentinel cats and pharyngeal and rectal swab specimens from 2 were virus positive by reverse transcription–PCR (cycle threshold >35) 2–6 days after first contact with infected cats. No virus was isolated from these swab specimens.

**Figure:**
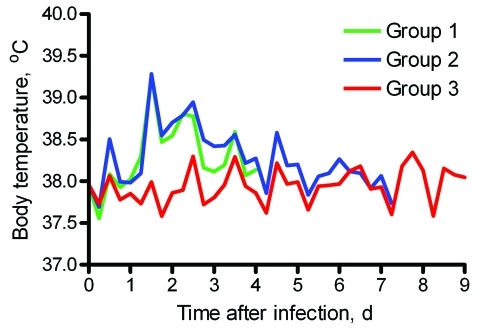
Average body temperatures of 2 groups of cats experimentally infected with pandemic (H1N1) 2009 virus (groups 1 and 2) and sentinel cats (group 3).

On 4 dpi, high virus titers were found in lungs, bronchi, and tracheas from 4 infected cats (10^5.5–6.3^, 10^2.9–4.6^, and 10^3.1–3.8^ TCID_50_/g, respectively). Tonsils from 2 cats, intestines from 1 cat, and the spleen from 1 cat also had high virus titers (10^3.0^, 10^4.2^, 10^1.6^, and 10^1.6^ TCID_50_/g, respectively). On 7 dpi, virus was detected in lung from 1 cat and trachea from 1 cat (10^3.0^ and 10^1.6^ TCID_50_/g, respectively). Infectious virus (10^1.2–2.2^ TCID_50_/g) was found in liver, intestine, brain, adrenal glands, and nictitating membranes of individual cats. The olfactory bulb of 1 cat was virus positive (10^3.0^ TCID_50_/g). No other organs from any cats were virus positive (Table).

**Table:** Test results for 8 cats intratracheally infected with pandemic (H1N1) 2009 virus*

Tissue source	No. positive				
	IHC analysis			Virus isolation†	
	4 dpi	7 dpi		4 dpi	7 dpi
Respiratory					
Lung	4	3		4	1
Bronchus	2	0		4	0
Trachea	0	0		4	1
Nasal turbinates	0	0		2	0
Extrarespiratory					
Liver	0	0		0	1
Intestine	0	0		1	1
Olfactory bulb	0	0		0	1
Brain	0	0		0	1
Spleen	0	0		1	0
Tonsil	0	0		2	0
Adrenal gland	0	0		0	2
Nictitating membrane	0	0		0	2

*Four cats were examined on each day. IHC, immunohistochemical; dpi, days postinfection. †No virus was isolated from tracheobronchial lymph node, pancreas, heart, or kidney of any cats.

No serum HI antibodies (titer <20) were found in group 1 cats on 4 dpi. All group 2 cats had serum HI antibodies (titers 30–120) on 7 dpi. One sentinel cat was seropositive on 15 dpi (titer 40); all cats were positive on 21 dpi (titer 80).

All infected cats showed multifocal or coalescing pulmonary consolidation, ranging from 30% to 50% on 4 dpi and from 10% to 30% on 7 dpi (Technical Appendix). All tracheobronchial lymph nodes were enlarged 3–5×. Palatine tonsils were enlarged ≈2× on 7 dpi. All sentinel cats showed mild multifocal consolidation; 5%–10% of lung parenchyma were affected. Two cats had tracheobronchial lymph nodes enlarged 2–5×.

Histopathologic analysis (Technical Appendix) identified pulmonary consolidation indicative of diffuse alveolar damage. Alveolar and bronchiolar lumina showed edema and contained variable numbers of macrophages, neutrophils, and erythrocytes mixed with fibrin and cellular debris. Alveolar walls were thickened and showed necrosis of lining epithelium and type II pneumocyte hyperplasia.

Bronchiolar walls were moderately infiltrated by neutrophils and had multifocal epithelial necrosis and multifocal peribronchiolar moderate infiltration by macrophages and lymphocytes and few neutrophils and plasmacytes. Bronchial lumina harbored few neutrophils and scant edema, fibrin, and cellular debris. There were few peribronchial infiltrates with a small number of lymphocytes, plasmacytes, and macrophages. Lung lesions seen on 4 dpi and 7 dpi were comparable except for more extensive type II pneumocyte hyperplasia on 7 dpi. Tracheobronchial lymph nodes and palatine tonsils had severe sinus histiocytosis and lymphocytolysis and moderate infiltration by neutrophils. Histologic changes in lung parenchyma of all sentinel cats were consistent with chronic lesions resulting from those seen in the other cats. No lesions were seen in other organs of all cats.

Virus antigen expression was more prominent on 4 dpi than on 7 dpi and was closely associated with histologic lesions (Technical Appendix). Virus antigen expression was seen in many type II pneumocytes, few type I pneumocytes, alveolar macrophages, bronchiolar ciliated and nonciliated epithelial cells, and rare bronchial ciliated epithelial cells. Type I and II pneumocytes were identified by double-staining with cytokeratin. No virus antigen was observed in sentinel cats.

## Conclusions

Intratracheal infection of domestic cats with pandemic (H1N1) 2009 virus resulted in mild-to-moderate clinical signs and virus replication throughout the respiratory tract, which caused diffuse alveolar damage. The pathogenesis in the respiratory tract in cats was similar to that occurring in humans, macaques, and ferrets (*7**,**11**–**13*). Seroconversion of sentinel cats indicated cat-to-cat transmission.

Unlike infection with seasonal human influenza viruses, infection with pandemic (H1N1) 2009 virus causes respiratory disease in cats. To compare infections with these viruses, we used our unpublished data for cats intratracheally infected with 10^5.0^ TCID_50_ of HPAI virus (H5N1) (A/Indonesia/5/05) at 4 dpi and 7 dpi (*4*) and for sham-infected cats. Histopathologic and immunohistochemical findings in lungs of cats infected with these viruses coincided, which indicated a similar pathogenetic process and increased severity in cats infected with HPAI virus (H5N1). However, in contrast to HPAI virus (H5N1), pandemic (H1N1) 2009 virus does not cause extrapulmonary lesions in infected cats. Our data show that pandemic (H1N1) 2009 virus may cause respiratory disease in cats and that human-to-cat transmission is the most likely route of infection.

## Supplementary Material

Technical Appendix Macroscopic, histopathologic, and immunohistochemical analysis on day 4 of lungs of infected cats.
