# Supplementary material for: Experimental Pandemic (H1N1) 2009 Virus Infection of Cats
Source: Emerg Infect Dis. 2010 Nov;16(11):1745–7. doi: 10.3201/eid1611.100845 (PMC3294532; doi:10.3201/eid1611.100845)
Supplement: Technical Appendix — Macroscopic, histopathologic, and immunohistochemical analysis on day 4 of lungs of infected cats. [file 10-0845-Techapp.pdf]

Article DOI: 10.3201/eid1611.100845

Experimental Pandemic (H1N1) 2009 Virus Infection of Cats

Technical Appendix

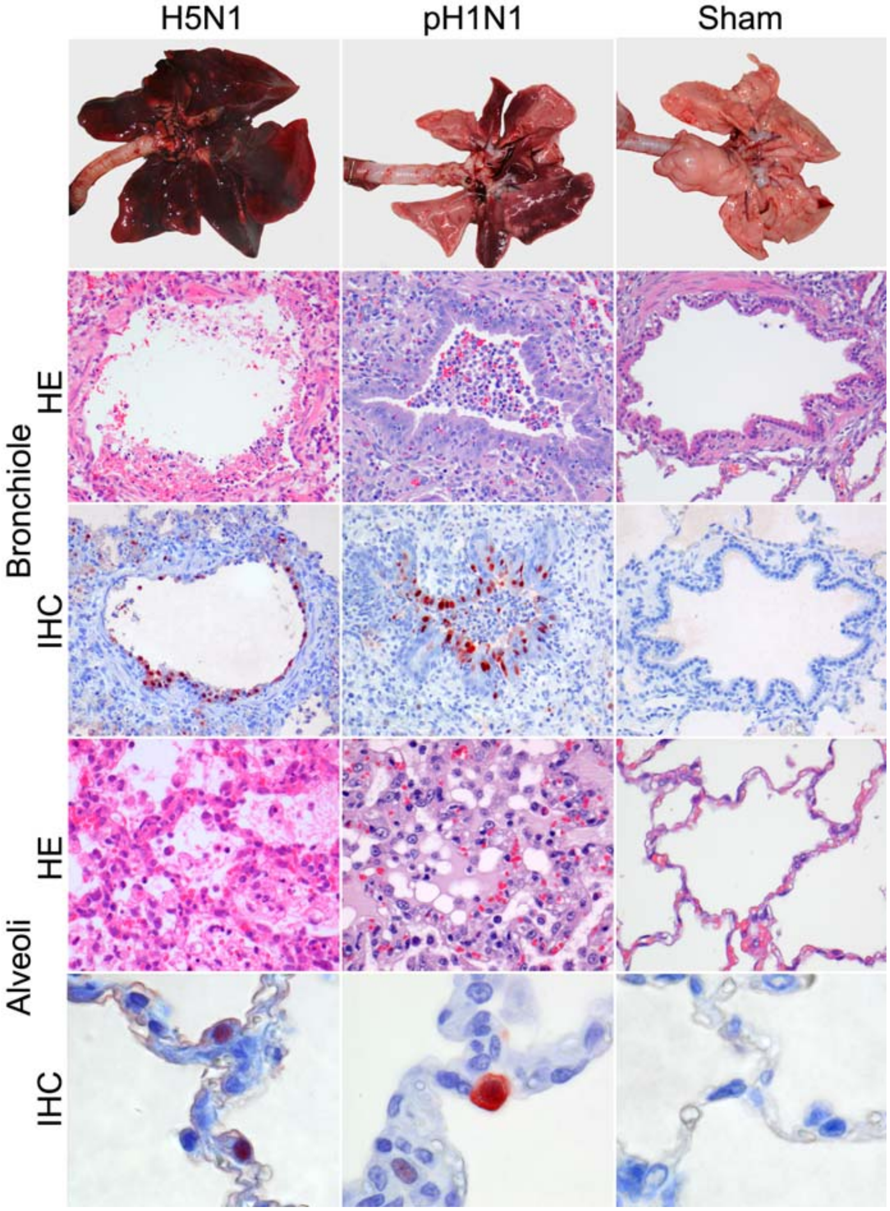

Macroscopic, histopathologic, and immunohistochemical analysis on day 4 of lungs of cats infected with highly pathogenic avian influenza (HPAI) virus (H5N1), pandemic (H1N1) 2009 virus (pH1N1), or phosphate-buffered saline (Sham). Macroscopic analysis of lungs (top row) in cats infected with HPAI

virus (H5N1), showing multifocal or coalescing consolidation characterized by dark red, firm, slightly raised areas. Multifocal consolidation occurred in the group infected with pandemic (H1N1) 2009 virus. The sham-infected group had no lesions. Lesions of cats infected with HPAI virus (H5N1) (first column), showing severe epithelial necrosis in bronchioles and alveoli (hematoxylin and eosin [HE] stain) with intraluminal edema and inflammatory cell infiltrates, and associated influenza virus antigen expression (immunohistochemical stain with 3-amino-9-ethylcarbazole substrate and hematoxylin counterstain [IHC]) in bronchiolar and type II alveolar epithelial cells. The group infected with pandemic (H1N1) 2009 virus (second column) had lesions characterized by inflammatory cell infiltrates in bronchioles and alveoli (HE stain) associated with influenza virus antigen expression in bronchiolar epithelial cells and type II alveolar epithelial cells (IHC stain). The group infected with phosphate-buffered saline (third column) showed no lesions (HE stain) or expression of influenza virus antigen (IHC stain). Original magnification, bronchiole x200, alveoli HE x400, and alveoli IHC x1,000.
